# High‐throughput liquid chromatography‐vacuum differential mobility spectrometry‐mass spectrometry for the analysis of isomeric drugs of abuse in human urine

**DOI:** 10.1002/dta.3778

**Published:** 2024-07-31

**Authors:** Maria Fernanda Cifuentes Girard, Patrick Knight, Gérard Hopfgartner

**Affiliations:** ^1^ Life Sciences Mass Spectrometry, Department of Inorganic and Analytical Chemistry University of Geneva Geneva 4 Switzerland; ^2^ Shimadzu Research Laboratory Manchester UK

**Keywords:** differential mobility spectrometry, liquid chromatography, mass spectrometry, urine drugs of abuse

## Abstract

The use of differential mobility spectrometry at low pressure coupled to liquid chromatography‐mass spectrometry (LC‐vDMS‐MS) was investigated for the analysis of 13 drugs of abuse (DoA) including the following: cocaine, ecgonine methyl ester, cocaethylene, benzoylecgonine, norcocaine, tramadol, isomeric pairs of metabolites; *O*‐desmethyl‐cis‐tramadol and *N*‐desmethyl‐cis‐tramadol, and cannabinoids: Δ^9^‐tetrahydrocannabinol, Δ^9^‐tetrahydrocannabidiol, 11‐hydroxy‐Δ^9^‐tetrahydrocannabinol, 11‐nor‐9carboxy‐Δ^9^‐tetrahydrocannabinol, and 11‐nor‐9carboxy‐Δ^9^‐tetrahydrocannabinol glucuronide. Different parameters were optimized for isomeric separation, such as LC mobile phase composition (20%–100% methanol acetonitrile and isopropanol, flow rate: 8–100 μL/min) and DMS separation voltage. Methanol and acetonitrile significantly affected the compensation voltage of the analytes and improved DMS separation. A short trap/elute LC‐vDMS‐SIM/MS screening method of 1 min was developed to quantify 11 drugs of abuse (except THC/CBD), in addition to a 4‐min LC‐vDMS‐SIM/MS method to identify and quantify five cannabinoids including the isomers THC/CBD and three THC metabolites. THC is the principal psychoactive constituent of cannabis and is a controlled substance in comparison to its isomeric counterpart CBD; this highlights the importance and challenges to resolve these isomeric pairs by analytical techniques. The signal responses were linear over a concentration range of 0.005–10 μg/mL for the DoA and 1–1000 ng/mL for cannabinoids. The intraday and interday precision were better than 12.2% and accuracy better than 115%. Urine samples from subjects who tested positive for THC and/or cocaine during roadside drug testing were evaluated to assess the performance of the methods LC‐vDMS‐SIM/MS and LC‐MRM/MS. Results show that the developed LC‐vDMS‐SIM/MS method presents similar performance to LC‐MRM/MS with improved sample throughput.

## Graphical abstract

This report describes the potential of vacuum differential mobility spectrometry (DMS) as an additional separation dimension to reduce liquid chromatography (LC‐MS) analysis time while maintaining good quantitative performance. A 1‐min trap/elute LC‐vDMS‐SIM/MS method for quantification of 11 drugs of abuse was developed and applied to the analysis of urine samples.

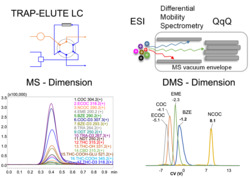

## 1 INTRODUCTION

In forensic toxicology, the identification and quantification of drugs of abuse and their metabolites in biological samples are crucial to confirm drug intake. Drugs of abuse (e.g., cocaine, tramadol, cannabinoids, morphine, and amphetamines) are controlled substances that are commonly abused or misused worldwide. The global number of substance users is expected to grow 25% by 2050, directly impacting road safety and public health^1^ with a concomitant increase in the requirement for testing. Many analytical procedures have been described to quantify commonly encountered drugs of abuse and their metabolites in biological matrices including saliva,^2^ sweat,^3^ hair,^4^ urine,^5^ and blood by liquid chromatography and gas chromatography coupled to mass spectrometry (LC‐MS, GC‐MS).^1^, ^6^, ^7^ Ion mobility (IMS) has drawn attention for the analysis of drugs of abuse, especially for isomeric metabolites, either as a standalone system or coupled to mass spectrometry (IMS‐MS). Drift time ion mobility‐time of flight (DTIMS‐TOF) derived collision cross section (CCS) has been used for the improved analysis of drugs of abuse (DoA) in blood samples.^8^ DTIMS has been applied for fast opioid profiling in human urine using flow‐injection analysis (FIA).^9^ Gwak et al.^10^ described a drift time ion mobility‐time of flight (DTIMS)‐TOFMS method for screening 35 new psychoactive substances (NPS) in seized drug samples. Additionally, Kanu et al.^11^ have reported an electrospray ionization atmospheric pressure ion mobility time‐of‐flight mass spectrometric method (APIMS‐TOFMS) for the analysis of psychoactive cathinones and tryptamines in less than 1 min. Tose et al.^12^ described a traveling wave ion mobility mass spectrometry (TWIM‐MS) method for isomeric separation of cannabinoids in hashish samples, marijuana, and parts of the *Cannabis sativa L*. plant (flower and leaf). Differential ion mobility (DMS) or high field asymmetric waveform ion mobility (FAIMS) exploits the ion mobility that occurs in the electric field in the region between two electrodes when an asymmetric waveform is applied orthogonal to the gas stream. Throughout the manuscript, we will use the acronym DMS. DMS is generally combined with triple quadrupole linear ion trap, quadrupole time of flight, or orbitrap, and it offers the possibility to add chemical modifiers (e.g., isopropanol, acetone, toluene) to the gas flow to tune separation selectivity and/or improve S/N ratio. Porta et al.^13^ described a modifier‐assisted differential ion mobility spectrometry‐mass spectrometry (DMS‐MS) hyphenated to liquid extraction surface (LESA) method for the analysis of 30 drugs of abuse and metabolites in human post‐mortem kidney and muscle tissues. Mashmoushi et al.^14^ reported the separation of isomeric cannabinoids including THC and CDB by DMS‐MS negative ion mode using isopropanol as modifier. A sensitive DMS‐MS/MS method was developed and validated for simultaneous identification of 33 drugs and metabolites in human urine samples, providing better analytical performance than immunoassay.^15^ Furthermore, DMS has been implemented to compensate for liquid chromatography selectivity loss in trap‐elute approaches and open port probe sampling interface for high‐throughput analysis. Gómez‐Ríos et al.^16^ reported a fast quantitation of opioids isomers in human plasma using biocompatible solid‐phase microextraction (bio‐SPME) and an open‐port probe sampling interface. Sosnowski et al.^17^ describe the application of an in‐house 3D‐printed open port probe (3DP‐OPP) with DMS‐MS for the quantitative analysis of cocaine and tramadol isomeric metabolites in urine samples and for the screening of illicit ecstasy pills using acetonitrile as modifier. While most DMS applications have been established on systems running at atmospheric pressure, a prototype FAIMS working at low pressure range of 4.7–30 Torr (vDMS) was recently developed, employing a planar‐gap stage within the MS instrument envelope rising normalized electric field up to 543 Td.^18^, ^19^ Compared with atmospheric DMS, besides the possibility to reach extreme fields, isolating of the DMS stage from the ion source allows the selection of the carrier gas without effecting the electrospray process.^18^ Girard et al.^20^ reported that in reported that in vDMS and at electrospray ionization flow rates >10 μL/min the mobile phase composition affects the compensation voltage (CV) which shifts to more negative values and improves the separation of isomeric and isobaric analytes. It was postulated that a small fraction of the solvent is getting into the mobility cell through the interface and is playing a role in the clustering‐declustering mechanism, even at very low concentrations.^20^ The effect of LC mobile phase, composition, and flow rate in vDMS is beneficial to improve the assay selectivity and reduce analysis time and was applied to the quantitative analysis of antidepressants in plasma^20^ and pyrrolizidine alkaloids in tea samples.^21^

This report describes the potential of vDMS as an additional separation dimension to reduce LC analysis time while maintaining good quantitative performance for the analysis of drug of abuse (DoA). A set of model compounds includes cocaine, ecgonine methyl ester, cocaethylene, tramadol, and isomeric pairs of metabolites such as benzoylecgonine, norcocaine, *O*‐desmethyl‐cis‐tramadol, and *N*‐desmethyl‐cis‐tramadol. Cannabinoids Δ^9^‐tetrahydrocannabinol, Δ^9^‐tetrahydrocannabidiol, 11‐hydroxy‐Δ^9^‐tetrahydrocannabinol, 11‐nor‐9carboxy‐Δ^9^‐tetrahydrocannabinol, and 11‐nor‐9carboxy‐Δ^9^‐tetrahydrocannabinol glucuronide were used to develop two assays in urine using a short LC column operated in trap/elute mode (LC‐vDMS‐SIM/MS) and detection in the selected ion monitoring mode. A 1‐min LC‐vDMS‐SIM/MS method for quantification of 11 drugs of abuse (excluding THC/CBD) was developed, in addition to a 4‐min method to confirm and quantify the cannabinoids THC, CBD, and metabolites. The performance of these assays was compared with liquid chromatography methods^22^ using the multiple reaction monitoring mode (LC‐MRM/MS).

## 2 EXPERIMENTAL

### 2.1 Materials and chemicals

Cocaine (COC), benzoylecgonine (BZE), norcocaine (NCOC), ecgonine methyl ester (EME), cocaethylene (ECOC), tramadol (TRA), *O*‐desmethyl‐cis‐tramadol (ODT), *N*‐desmethyl‐cis‐tramadol (NDT), Δ^9^‐tetrahydrocannabinol (THC), Δ^9^‐tetrahydrocannabidiol (CBD), 11‐hydroxy‐Δ^9^‐tetrahydrocannabinol (THC‐OH), 11‐nor‐9carboxy‐Δ^9^‐tetrahydrocannabinol (THC‐COOH), and 11‐nor‐9carboxy‐Δ^9^‐tetrahydrocannabinol glucuronide (THC‐COOH‐GLU) were purchased from Lipomed (Arlesheim, Switzerland). Internal standards cocaine‐D_3_ (COC‐D_3_), benzoylecgonine‐D_3_ (BZE‐D_3_), tramadol‐D_3_ (TRA‐D_3_), and Δ^9^‐tetrahydrocannabinol‐D_3_ (THC‐D_3_) were purchased from Lipomed (Arlesheim, Switzerland). For structures, see Figure S1.

LC‐MS grade acetonitrile was obtained from VWR Chemicals (Fontenay‐sous‐Bois, France) and methanol and formic acid from Fischer Scientific AG (Reinach, Switzerland). UHPLC‐MS grade water was obtained from Huberlab (Aesch, Switzerland).

### 2.2 Sample preparation

#### 2.2.1 Preparation of standards solutions

Stock solutions of drugs of abuse were prepared by dissolving each standard in MeOH at 1 mg/mL. The working solutions were diluted to the desired concentration in 50/50 MeOH/H_2_O, 0.1% formic acid.

#### 2.2.2 Urine sample preparation

Urine samples from control subjects and subjects who tested positive for THC and/or cocaine during roadside drug testing were provided by the Institute of Forensic Medicine from the University of Bern, Switzerland. All samples were provided as anonymized biological materials; hence, the Swiss Human Research Act (HRA) did not apply (Article 2.2. b). Aliquots of the samples were stored in the freezer at −20°C. Prior to analysis, 100 μL of urine samples were thawed, fortified with 400 ng/mL IS (cocaine‐D_3_, benzoylecgonine‐D_3_, tradamol‐D_3_ and THC‐D_3_), vortexed briefly, and centrifuged for 10 min at 14000 rpm at 4°C.

#### 2.2.3 Calibration and quality control samples

Calibration curves were fortified in pooled human control urine samples to final concentrations of 5, 50, 250, 500, 1000, 5000, and 10,000 ng/mL and internal standard concentration of 400 ng/mL for cocaine, tramadol, and their metabolites. The pooled human urine were drug free samples previously analyzed (data not shown). LLOQ, QCLow, QCMedium, and QCHigh were prepared independently at four concentrations (5, 15, 3800, and 7500 ng/mL). For cannabinoids, calibration curves were from 1, 10, 100, 250, 500, and 1000 ng/mL. LLOQ, QCLow, QCMedium, and QCHigh were prepared independently at four concentrations (1, 3, 375, and 750 ng/mL). Blank urine (pooled urine control sample with internal standard) was run as well with each analytical batch. Assay linearity, accuracy, precision, detection limit, and quantitation limit were evaluated to ensure the assay was fit for purpose.^23^ These parameters were determined for both methods; five replicate analyses were performed during three nonconsecutive days.

### 2.3 Liquid chromatography‐mass spectrometry

#### 2.3.1 ESI‐LC‐vDMS‐SIM/MS analysis

Liquid chromatography was performed on a Nexera Mikros LC system (Shimadzu Corporation, Kyoto, Japan) composed of one LC‐30AD pump, an LC‐Mikros microflow pump, a SIL‐30AC autosampler, a six‐port switching valve FCV‐32AH, and a CTO‐Mikros column oven with UF‐Link. The UHPLC system was coupled to an LCMS‐8060 triple quadrupole mass spectrometer equipped with an ESI source and a prototype vDMS cell operating at a pressure of 33 mbar (Shimadzu Research Laboratory, UK). The operating gas flows were the following: nebulizing gas (NB) flow 1.5 L/min (N2), drying gas (DG) OFF, heating gas flow OFF (Air), CID gas 17 kPa, interface voltage 2 kV and temperature 200°C, DL temperature 200°C, heat block temperature 400°C. For trap‐elute LC, an additional pump (LC‐30AD) was used and a Reprosil–Pur C18‐AQ (10 × 0.5 mm, 5 μm, Dr. Maisch GmbH, Germany) was mounted on a six‐port switching valve as illustrated in Figure S2. In a first step, analytes were retained on the column (25°C) using a mixture of H_2_O/CH_3_CN (90/10; v/v), 0.1% formic acid at 100 μL/min. After 0.25 min, the valve was switched and the analytes eluted in back‐flush mode to the mass spectrometer using H_2_O/CH_3_CN (80/20; v/v), 0.1% formic acid at 100 μL/min. The total run time was 1 min with an injection volume of 10 μL. Data were acquired and processed using LabSolutions (version 5.99 SP2, Shimadzu Corporation, Kyoto, Japan). Table 1 summarizes the LC, MS, and vDMS optimized parameters for the analysis of eight drugs of abuse and five cannabinoids.

**TABLE 1:** MRM, SIM, and DMS parameters of drug of abuse and their metabolites (*n* = 13).

Compounds	LC‐MRM/MS					Short LC‐DMS‐SIM/MS			
	Q1 (*m/z*)	Q3 (*m/z*)	CE (V)	RT (min)	IS	RT (min)	IS	CV (V)	DV (V)
Cocaine (COC)	304.1	182.3	22	2.87	COC‐D_3_	0.42	COC‐D_3_	−4.1	600
		82.2	31						
Benzoylecgonine (BZE)	290.1	168.2	19	1.73	BZE‐D_3_	0.43	BZE‐D_3_	−1.2	600
		83.2	35						
Norcocaine (NCOC)	290.1	136.1	20	2.59	COC‐D_3_	0.42	COC‐D_3_	8.1	600
		168.2	15						
Ecgonine methyl ester (EME)	200.1	182.2	18	3.80	COC‐D_3_	0.41	COC‐D_3_	−2.3	600
		82.1	23						
Cocaethylene (ECOC)	318.2	196.3	21	3.09	COC‐D_3_	0.41	COC‐D_3_	−5.1	600
		82.1	30						
Tramadol (TRA)	264.1	58.1	30	3.83	TRA‐D_3_	0.41	TRA‐D_3_	−4.9	600
		91.1	35						
*O*‐desmethyl‐cis‐tramadol (ODT)	250.1	58.2	22	3.70	TRA‐D_3_	0.42	TRA‐D_3_	−2.9	600
*N*‐desmethyl‐cis‐tramadol (NDT)	250.1	44.1	35	4.81	TRA‐D_3_	0.42	TRA‐D_3_	8.2	600
Δ^9^‐tetrahydrocannabinol (THC)	315.2	123.2	43	3.10	THC‐D_3_	0.43	THC‐D_3_	5.9	600
		193.1	26						
Δ^9^‐tetrahydrocannabidiol (CBD)	315.2	193.1	23	2.35	THC‐D_3_	0.43	THC‐D_3_	5.3	600
		123.2	43						
11‐Hydroxy‐Δ^9^‐tetrahydrocannabinol (THC‐OH)	331.1	91.1	41	1.95	THC‐D_3_	0.42	THC‐D_3_	4.4	600
		67.2	54						
11‐nor‐9carboxy‐Δ^9^‐tetrahydrocannabinol (THC‐COOH)	345.2	57.2	55	1.85	THC‐D_3_	0.42	THC‐D_3_	−6.7	600
		119.2	34						
11‐nor‐9carboxy‐Δ^9^‐tetrahydrocannabinol glucuronide (THC‐COOH‐GLU)	521.2	352.2	40	1.15	THC‐D_3_	0.42	THC‐D_3_	−8.0	600
		89.2	35						
Cocaine‐D_3_ (COC‐D_3_)	307.3	185.3	19	2.94	N.A.	0.42	N.A.	−4.2	600
		85.1	30						
Benzoylecgonine‐D_3_ (BZE‐D_3_)	293.1	171.3	20	1.73	N.A.	0.43	N.A.	−1.3	600
		77.2	35						
Tramadol‐D_3_ (TRA‐D_3_)	267.1	58.3	40	3.83	N.A.	0.41	N.A.	−4.8	600
		91.1	35						
Δ^9^‐tetrahydrocannabinol‐D_3_ (THC‐D_3_)	318.2	196.3	22	3.10	N.A.		N.A.	5.8	600
		123.1	35						

#### 2.3.2 ESI‐LC‐MRM/MS analysis

Liquid chromatography (conditions as reported by Zheng et al.^22^) was performed on a Nexera UHPLC (Shimadzu Corporation, Kyoto, Japan) composed of one LC‐30AD pump, a SIL‐30AC autosampler, and a CTO‐30A oven. The UHPLC system was coupled to an LCMS‐8060 triple quadrupole mass spectrometer equipped with an ESI source and a prototype vDMS cell operating at a pressure of 33 mbar (Shimadzu Research Laboratory, UK). The nebulizing gas flow was of 1.5 L/min (N_2_), the drying gas flow of 3.0 L/min (N_2_), and the heating gas flow of 3.0 L/min (Air). The interface voltage was 2 kV and temperature 200°C, the DL temperature of 200°C, and the heat block temperature of 400°C. For LC‐MRM/MS analysis, Argon was used as CID gas (270 kPa) and the vDMS was switched off.

For the analysis of the cocaine, tramadol, and their metabolites the analytical column was an Acquity UPLC BEH‐C18 column (3.0 × 100 mm, 1.7 μm, Waters). The column temperature was set at 50°C. Mobile phase A was aqueous 0.1% ammonium hydroxide and mobile phase B methanol. The gradient started at 10% B and a linear profile curve was used for the change in mobile phase composition followed by re‐equilibration time of 2 min (10% B). The total LC run time was 7 min. The flow rate was set at 0.6 mL/min and injection volume to 10 μL. Data were acquired and processed using LabSolutions (version 5.99 SP2, Shimadzu Corporation, Kyoto, Japan). Table 1 summarizes the LC and MS optimized parameters for the analysis of eight drugs of abuse and five cannabinoids.

#### 2.3.3 LC‐MRM/MS and LC‐vDMS‐SIM/MS analysis of cannabinoids

For the cannabinoids, the analytical column was a Kinetex XB‐C18 column (50 × 1.0 mm, 2.6 μm, Phenomenex). The column temperature was set at 40°C. Mobile phase A was H_2_O with 0.1% HCOOH and mobile phase B: CH_3_CN with 0.1% HCOOH. The ballistic gradient started at 77% B and was linearly increased to 95% B from 0.5 to 3 min and kept constant for 0.5 min (95% B) followed by re‐equilibration time of 1 min (10% B). The total LC run time was of 4 min. The flow rate was set at 0.1 mL/min and injection volume to 10 μL. For vDMS‐SIM/MS, the same settings were applied as shown in Section 2.3.1. For LC‐MRM/MS analysis, Argon was used as CID gas (270 kPa) and the vDMS was switched off. Data were acquired and processed using LabSolutions (version 5.99 SP2, Shimadzu Corporation, Kyoto, Japan). Table 2 summarizes the LC, MRM, and DMS parameters of five cannabinoids.

**TABLE 2:** MRM, SIM and DMS parameters of cannabinoids and metabolites (*n* = 5).

Compounds	LC‐MRM/MS					LC‐DMS‐SIM/MS			
	Q1 (*m/z*)	Q3 (*m/z*)	CE (V)	RT (min)	IS	RT (min)	IS	CV (V)	DV (V)
Δ^9^‐tetrahydrocannabinol (THC)	315.2	123.3	35	3.24	THC‐D_3_	3.24	THC‐D_3_	8.3	600
		193.3	35						
Δ^9^‐tetrahydrocannabidiol (CBD)	315.2	57.2	35	2.51	THC‐D_3_	2.51	THC‐D_3_	8.4	600
		123.2	35						
11‐Hydroxy‐Δ^9^‐tetrahydrocannabinol (THC‐OH)	331.2	91.2	50	2.30	THC‐D_3_	2.30	THC‐D_3_	7.6	600
		67.3	50						
11‐nor‐9carboxy‐Δ^9^‐tetrahydrocannabinol (THC‐COOH)	345.2	57.2	35	2.27	THC‐D_3_	2.27	THC‐D_3_	2.6/4.5	600
		119.2	35						
11‐nor‐9carboxy‐Δ^9^‐tetrahydrocannabinol glucuronide (THC‐COOH‐GLU)	521.2	89.2	35	1.05	THC‐D_3_	1.05	THC‐D_3_	−9.1	600
		91.2	35						
Δ^9^‐tetrahydrocannabinol‐D_3_ (THC‐D_3_)	318.2	197.3	35	3.4	N.A.	3.4	N.A.	8.1	600
		123.1	35						

## 3 RESULTS AND DISCUSSION

### 3.1 LC solvent composition effect on resolution of isomeric drugs of abuse

**FIGURE 1:**
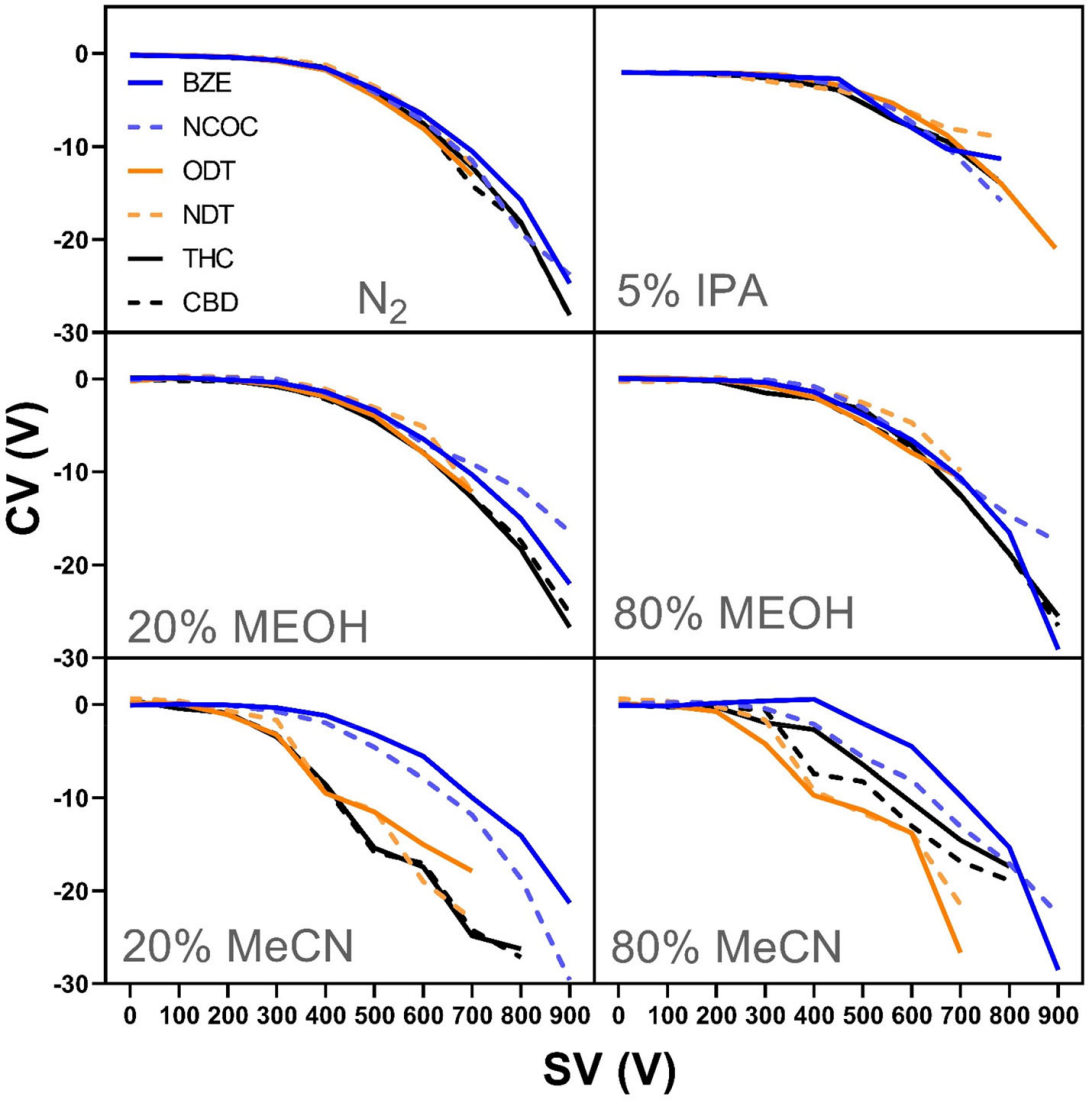
Dispersion plots (CV vs. SV) for the isomeric pairs of drugs of abuse (m/z 290) BZE/NCOC, (m/z 250) ODT/NDT, and (m/z 315) THC/CBD. (a) Infusion at 8 μL/min and in (b–f) flow injection analysis at flow rate 50 μL/min with 5% isopropanol, 20% and 80% methanol, and acetonitrile. Data were acquired in SIM mode, and the vDMS was used in scan mode (SV ramp from 0 to 900 V and CV stepped by 0.2 V).

It has been previously reported that at LC flow rates >10 μL/min, the mobile phase composition affects, in vDMS, the CV of the molecules and this effect can be used to optimize the DMS separation of isobars or isomers.^20^, ^21^ The DMS behavior at low pressure of three isomeric pairs, (*m/z* 290) BZE/NCOC (major metabolites of cocaine), (*m/z* 250) ODT/NDT (main metabolites of tramadol), and (*m/z* 315) THC/CBD were investigated. Six different gas‐phase environment conditions were investigated (different flow rates and mobile phase conditions), and the dispersion plots, compensation voltage (CV) vs. separation voltage (SV), are shown in Figure 1.

At a low flow rate of 8 μL/min (Figure 1a), the mobile phase composition should have almost no effect and peak capacity (PC) increases with separation voltage (SV) from 300 to 800 V (PC = 0.08 to PC = 0.63) under nitrogen conditions. Figure 1a displays a type C behavior, where an increasingly positive CV is required to correct ion trajectory with increasing SV, typical of hard sphere collisions. This gain in peak capacity at higher SV enables baseline separation of NCOC from BZE with resolution of 1.26 at SV of 800 V (198 Td). Regarding the tramadol metabolites, peak capacity (PC) increases from 300 to 700 V (PC 0.1 to 0.46) enabling partial resolution (RS) between the isomeric pair (RS 0.93), but above SV 700 V signal vanishes. For the isomeric pair THC/CBD (*m/z* 315), the peak capacity increases from PC = 0.01 to PC = 0.27; unfortunately, it was not sufficient to resolve the isomeric cannabinoids even at high electric field strength (E/N) of 223 Td (RS = 0.12). See Figure S3 for a more detailed view of the separations, presented as CV versus signal plots. Near baseline separation of Δ^9^‐THC, CBD was demonstrated on an atmospheric DMS device (SelexIon, Sciex) using isopropanol as modifier but requires at high values of SV = 4250 V close to electrical breakdown.^14^

Furthermore, the LC solvent effect on resolution of the isomeric pairs was investigated using methanol (20% and 80%), acetonitrile (20% and 80%), and 5% isopropanol. The compounds were analyzed by flow injection analysis (FIA) at flow rate 50 μL/min. With methanol, acetonitrile, and isopropanol, CV values shift to more negatives values as SV increased (several compounds reach −29 V at SV = 900 V) typical for type A behavior. Increasingly negative CV values with increasing SV are associated with strong interactions between the analyte ions and the carrier gas (solvent vapor) under low‐field conditions. Regarding the organic content of methanol in the mobile phase, CV values shift to more negative using 80% methanol compared with 20% methanol (7 V difference for THC/CBD), but this was insufficient to baseline resolve these isomers. When using acetonitrile, the best resolution (ΔCV = 8.3) for both BZE/NCOC and ODT/NDT was obtained at 20% acetonitrile and this condition was retained for further optimization.

**FIGURE 2:**
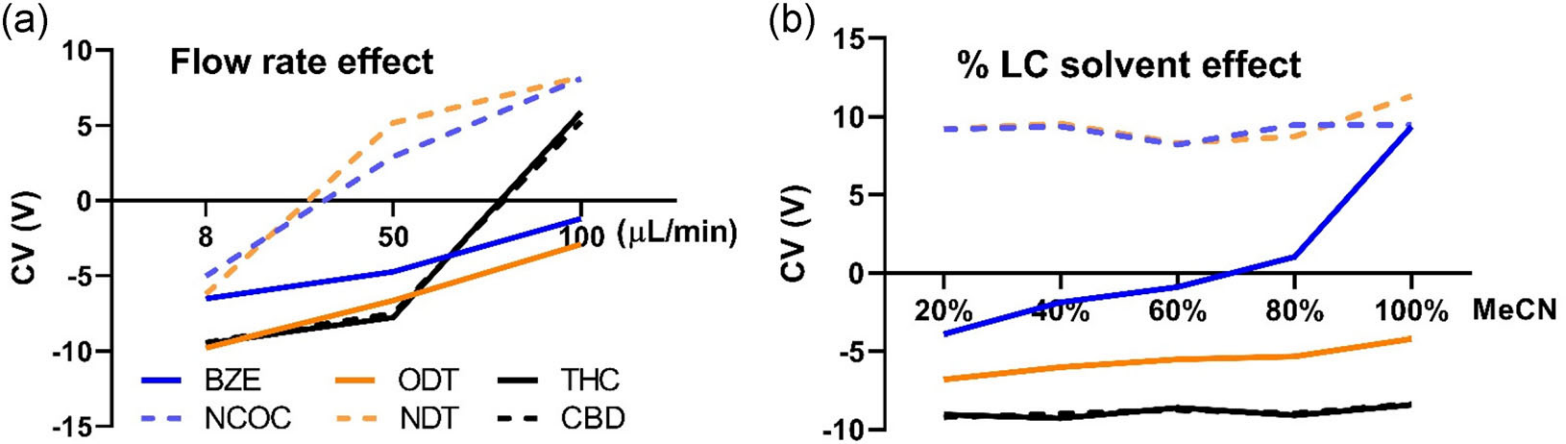
Effect of the increased flow rate, from 8 to 100 μL/min using 20% CH_3_CN (a) and CH_3_CN content, from 20% to 100% at 100 μL/min flow rate (b) for the resolution of isomeric pairs BZE/NCOC and ODT/NDT. vDMS was used in scan mode at SV = 600 V and CV stepped by 0.2 V.

**FIGURE 3:**
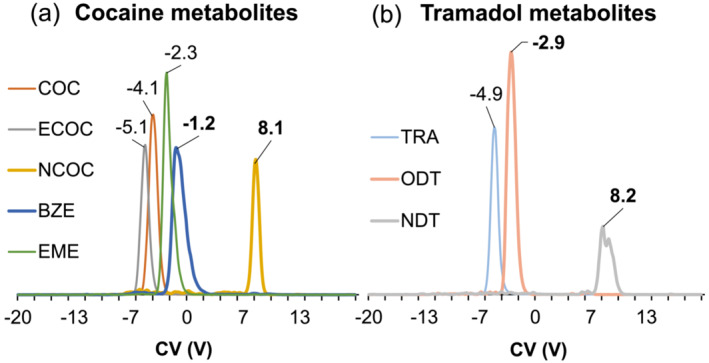
Measured compensation voltage (CV) at SV 600 V by FIA of cocaine and its metabolites including the isomeric metabolites BZE/NCOC (a) and tramadol and its isomeric metabolites ODT/NDT (b) at concentration of 500 ng/mL in SIM mode. Vacuum DMS was used in scan mode (CV stepped by 0.2 V) for optimized separation using 20% CH_3_CN on LC mobile phase at flow rate 100 μL/min.

Flow rates were varied from 8 to 100 μL/min and CV was scanned at SV 600 V. CV values shift as flow rates are modified; 100 μL/min was found to be the optimal for resolving the isomeric pairs BZE/NCOC and ODT/NDT (Figure 2a). As shown above, acetonitrile provides better resolution of the isomeric pairs, so the effect of varying the content of acetonitrile was investigated for resolution (Figure 2b). CV values slightly change as a function of the % acetonitrile, except for BZE for which the CV values became more positive as the % acetonitrile increased, eventually reaching the same CV value as NCOC (10 V), its isomeric counterpart. Overall, 20% acetonitrile and 100 μL/min were found to be optimal for resolving the isomeric pairs. Subsequently, CV was scanned using the optimized DMS conditions; cell temperature 60°C, pressure 33 mbar, F value 2, SV 600 V, flow rate 100 μL/min, and 20/80 ACN/H_2_O + 0.1% FA as mobile phase. Figure 3 presents the ionograms with CV values for optimized separation of cocaine, tramadol, and their metabolites. Under these optimized conditions, the isomeric pairs are baseline separated; the resolution between the isomers BZE/NCOC was RS = 27.34 and PC = 13.63. For the isomers, ODT/NDT resolution was RS = 8.51 and PC = 4.24.

### 3.2 Method performance

#### 3.2.1 Short liquid chromatography with selected ion monitoring (vDMS‐SIM/MS) using trap‐elute setup for high throughput analysis of drugs of abuse in urine samples

The optimized vDMS conditions were combined with a short column trap‐elute LC method. Analytes were trapped on a 1‐cm C18 column for 15 s using 10% acetonitrile and eluted in back‐flush mode using 20% acetonitrile, for a total run time of 1 minute as described in Figure S2. Compared with flow injection analysis, the trap‐elute method reduces ionization suppression by separating compounds based on physicochemical properties (pKa, Log P) in the condensed phase, while the vDMS dimension provides separation of target species based on gas phase interactions. The advantage compared with LC‐MS methods is seen in the reduced analysis time while maintaining sensitivity. Figure S4A,B illustrates the noise reduction in SIM mode obtained with the vDMS.

**FIGURE 4:**
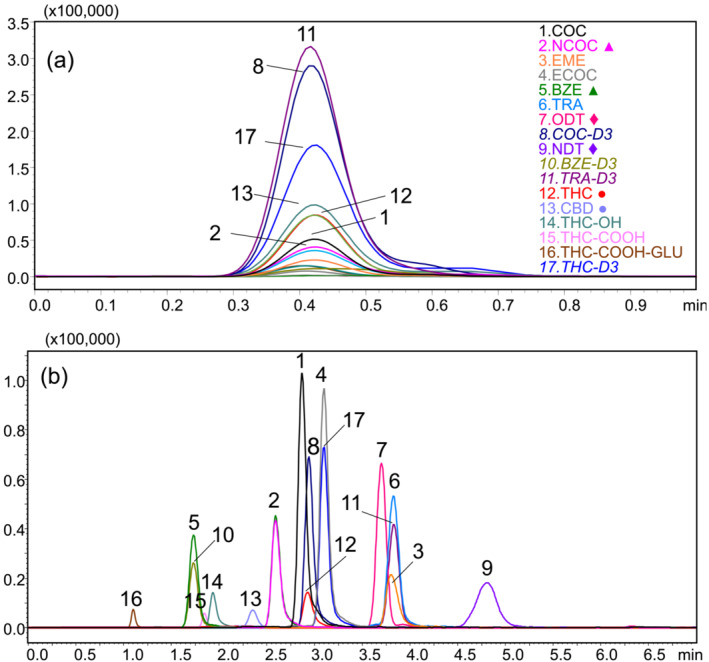
Representative extracted ion chromatogram of drugs of abuse (500 ng/mL) by short LC‐DMS‐SIM/MS, all compounds coeluted at 0.4 min (a) and (b) by LC‐MRM/MS reference method, ♦, ●, ▲ represent isomeric pairs.

The isomeric pairs benzoylecgonine, norcocaine (*m/z* 290), *O*‐desmethyl‐cis‐tramadol, and *N*‐desmethyl‐cis‐tramadol (*m/z* 250) coeluted in the LC dimension at 0.4 min as shown in Figure 4a, while in the DMS dimension, the isomers are baseline separated based on the interactions with acetonitrile from the LC mobile phase as shown in Figure 3b. These conditions provide the best resolution for the isomers BZE‐NCOC (RS = 12.14) and ODT‐NDT (RS = 11.52) and increase the analysis throughput by a factor of five compared with the LC‐MS/MRM method. vDMS also substantially reduces the chemical noise observed in blank urine samples by filtering the potential interferences when the potential is applied (Figure S4).

By this method, the presence of cannabinoids was identified but quantification of THC and CBD was not possible as these isomers were not resolved under the conditions investigated. An LC‐vDMS‐MS second method was developed to confirm the presence of isomeric THC/CBD and quantification of five cannabinoids in selected ion monitoring mode.

#### 3.2.2 LC‐vDMS‐MS using short gradient for quantification of cannabinoids in urine samples

**FIGURE 5:**
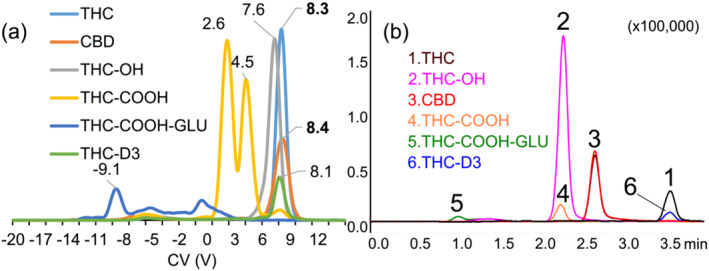
Representative overlaid ionograms of cannabinoids (500 ng/mL). (a) CV scans at SV = 600 V of each standard using a gradient 77%–95% acetonitrile as mobile phase at flow rate 100 μL/min. Peak CVs are indicated (b) extracted ion chromatogram of cannabinoids (500 ng/mL) by short gradient LC‐DMS‐SIM/MS. Retention times in minutes THC (3.24), CBD (2.51), THC‐OH (2.30), THC‐COOH (2.28), THC‐COOH‐GLU (1.05), THC‐D3 (3.24).

For the resolution of isomers THC and CBD (*m/z* 315), DMS conditions were optimized regarding LC solvent type, concentration (MeOH, ACN; IPA), and flow rate (8–100 μL/min). It was found that a short gradient (4 min) from 77% and 95% acetonitrile at 100 μL/min gave the optimum results. Figure 5a shows the overlaid CV values at SV 600 V for the cannabinoids; under these conditions, the isomers THC/CBD overlap at a CV 8.3 V. In contrast, in the LC dimension, the isomers are completely resolved at retention times 3.24 min for THC and 2.51 min for CBD as show in Figure 5b.

#### 3.2.3 Comparison in method performance using LC‐MRM/MS and short LC‐vDMS‐SIM/MS

The selected drugs of abuse and cannabinoids were fortified into a pooled analyte free urine sample to evaluate assay performance. The internal standards cocaine‐D3, tramadol‐D3, and THC‐D3 (400 ng/mL) were used to correct potential matrix effect and losses in sample preparation. For all DoA compounds, the assay response was found to be linear over more of three orders of magnitude, ranging from 5 to 10,000 ng/mL. Precision and accuracy were determined at four levels LLOQ (5 ng/mL), LQC (15 ng/mL), MQC (3800 ng/mL), and HQC (7500 ng/mL) for short LC‐vDMS‐SIM/MS (see Table S1) and LC‐MRM/MS (see Table S2). For the quantification of cannabinoids, the assay linearity response was from 1 to 1000ng/mL. The QC levels, LLOQ (1 ng/mL), LQC (3 ng/mL), MQC (375 ng/mL), and HQC (750 ng/mL) were evaluated for short gradient LC‐vDMS‐SIM/MS (see Table S3) and LC‐MRM/MS (see Table S4). For all methods, interassay accuracy was in the range of 93.9%–114% for all analytes (98.3%–115% at LLOQ) and interassay precision was lower than 12.2%. These findings imply that when compared with LC‐MRM/MS, the short LC‐vDMS‐SIM/MS technique performs similarly in terms of linearity, accuracy, and precision.

### 3.3 Application to urine samples collected during roadside drug testing

The three methods (2× LC‐vDMS‐SIM/MS and 1× LC‐MRM/MS) were applied to the quantification of 13 drugs of abuse in 15 urine samples. The urine samples were collected from subjects who tested positive for THC and/or cocaine during roadside drug testing. Samples were fortified with internal standards (cocaine‐D3, benzoylecgonine‐D3, tradamol‐D3, and THC‐D3 at 400 ng/mL) and centrifuged. Ten urine samples were first analyzed by 1‐min LC‐vDMS‐SIM/MS method for quantification of eight drugs of abuse/metabolites and qualitative identification performed for five cannabinoids/metabolites. Next, the second longer method of 4 min was performed to confirm and quantify the cannabinoids THC, CBD, and metabolites. Samples were reanalyzed by the reference LC‐MRM/MS method.

**FIGURE 6:**
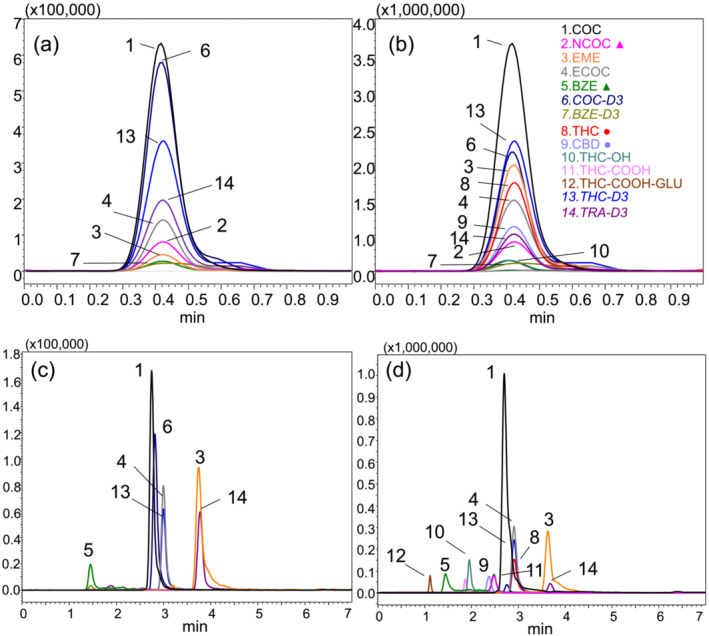
Extracted ion chromatogram of urine samples (a) by LC‐vDMS‐SIM/MS with positive results for cocaine but negative to THC/CBD, (b) by LC‐vDMS‐SIM/MS with positive results for cocaine and THC/CBD, (c) by LC‐MRM/MS with positive results for cocaine but negative to THC/CBD, and (d) by LC‐MRM/MS with positive results for cocaine and THC/CBD. ●, ▲ represent isomeric pairs.

Figure 6a shows a chromatogram for a urine sample positive for cocaine but negative for cannabinoids. In contrast, Figure 6b shows a urine positive for cocaine and cannabinoids by 1‐min LC‐vDMS‐SIM/MS method. The reference LC‐MRM/MS method produced similar results with chromatograms presented in Figure 6c,d.

The concentration of the 13 drugs of abuse and cannabinoids were calculated based on the calibration curve corrected by internal standards. Figure S5 shows the concentrations found in positive urine samples for cocaine and metabolites, concentration ranging from 472.5 to 12,963 ng/mL for cocaine. The parent drug cocaine can be easily hydrolyzed to BZE (2668–17,379 ng/mL) or EME (5247–23,962 ng/mL), even from an external source of contamination. Therefore, the detection of metabolites NCOC and ECOC formed by a different pathway is required to confirm the drug intake. ECOC was in the range of 48.2–2104 ng/mL; this particular toxic metabolite is formed with the simultaneous consumption of alcohol. NCOC was found ranging from 136.5 to 736.2 ng/mL and was distinguished from its isomeric pair BZE in the DMS dimension with a resolution of 12.4 under the optimized LC and DMS conditions.

Regarding the cannabinoids, Figure S6 shows the concentrations found in positive urine samples for cannabinoids and metabolites, concentration ranging from 4.905 to 40.52 ng/mL for THC. The isomeric CBD was baseline separated from THC in the LC dimension and was found on the range of 1.571–828.6 ng/mL. The metabolites that confirm the consumption of cannabis were found in the urine samples in the range of 1.203–93.36 ng/mL for THC‐OH, 21.71–1160 ng/mL for THC‐COOH, and 43.13–3490 ng/mL for THC‐COOH‐GLU (when measured by LC‐vDMS‐SIM/MS). Results of the reanalysis of the samples by the reference LC‐MRM/MS method are presented in Figures S5 and S6. The bias observed between the LC‐vDMS‐SIM/MS and LC‐MRM/MS methods was in the range −15% to +13% for all analytes.

## 4 CONCLUSIONS

In this work, two methods combining a short C18 column with trap/elute LC setup hyphenated to vDMS and mass spectrometric detection in SIM mode were developed for the simultaneous quantification of cocaine, tramadol, THC, CBD and their metabolites. A 1‐min LC‐vDMS‐SIM/MS method facilities screening of 13 drugs of abuse and quantification 12 (i.e., cocaine, tramadol, and their metabolites and five cannabinoids). A second 4‐min LC‐vDMS‐SIM/MS method was developed for subsequent accurate quantification of THC and CBD where detected. An advantage of the prototype vDMS system was that a small fraction of the LC organic solvent is transferred into the vDMS cell through the interface and works as a modifier in a cluster/declustering mechanism without the use of additional hardware. This characteristic enables optimization of vDMS performance via LC mobile phase conditions. In this case, the use of 20% acetonitrile as a modifier enables complete baseline separation of isomeric cocaine and tramadol metabolites facilitating a fivefold reduction in the analysis time compared with the LC‐MRM/MS method. In addition, vDMS can be used to remove potential interferences for improved quantification and selectivity. In the case of cannabinoids, the isomeric THC and CBD were separated in the LC dimension using a small gradient (77%–95% CH_3_CN) in SIM mode. The LC‐vDMS‐SIM/MS methods were compared with an LC‐MRM/MS method, and in all cases, interassay precisions were lower than 12.2% and interassay accuracy better than 115%, despite the reduced analysis times in the LC‐vDMS‐SIM/MS methods. Compared with flow injection analysis approach, the LC dimension remains important as it allows preconcentration of the analytes and reduces matrix effects demonstrating the benefit of LC and DMS for developing high‐throughput methods as applied here for DoA analysis.

## Supporting information


**Figure S1:** Chemical structures of drugs of abuse analytes.
**Figure S2**: Short LC‐vDMS‐MS configuration.
**Figure S3**: Overlaid compensation voltage plots for the isomeric pairs BZE/NCOC, ODT/NDT and THC/CBD at SV of 300 V.
**Figure S4**. Representative extracted ion chromatogram of urine sample acquired by LC‐vDMS‐SIM/MS.
**Figure S5**: Quantification of cocaine and metabolites.
**Figure S6**. Quantification of cannabinoids THC, CBD, and metabolites (THC‐OH, THC‐COOH, THC‐COOH‐GLU).
**Table S1**: Short LC‐DMS‐SIM/MS method performance for 8 drug of abuse and their metabolites in urine samples.
**Table S2**: LC‐MRM/MS method performance for 8 drug of abuse and its metabolites in urine samples.
**Table S3**: LC‐vDMS‐SIM/MS method performance for 5 cannabinoids in urine samples.
**Table S4**. LC‐MRM/MS method performance for 5 cannabinoids in urine samples.
