# Supplementary material for: High‐throughput liquid chromatography‐vacuum differential mobility spectrometry‐mass spectrometry for the analysis of isomeric drugs of abuse in human urine
Source: Drug Test Anal. 2024 Jul 31;17(6):751–60. doi: 10.1002/dta.3778 (PMC12151711; doi:10.1002/dta.3778)
Supplement: Supplementary file 1 — Figure S1: Chemical structures of drugs of abuse analytes. Figure S2: Short LC‐vDMS‐MS configuration. Figure S3: Overlaid compensation voltage plots for the isomeric pairs BZE/NCOC, ODT/NDT and THC/CBD at SV of 300 V. Figure S4. Representative extracted ion chromatogram of urine sample acquired by LC‐vDMS‐SIM/MS. Figure S5: Quantification of cocaine and metabolites. Figure S6. Quantification of cannabinoids THC, CBD, and metabolites (THC‐OH, THC‐COOH, THC‐COOH‐GLU). Table S1: Short LC‐DMS‐SIM/MS method performance for 8 drug of abuse and their metabolites in urine samples. Table S2: LC‐MRM/MS method performance for 8 drug of abuse and its metabolites in urine samples. Table S3: LC‐vDMS‐SIM/MS method performance for 5 cannabinoids in urine samples. Table S4. LC‐MRM/MS method performance for 5 cannabinoids in urine samples. [file DTA-17-751-s001.pdf]

Supplementary Information

High-Throughput Liquid Chromatography- Vacuum Differential Mobility Spectrometry-Mass Spectrometry for the Analysis of Isomeric Drugs of Abuse in Human Urine

Maria Fernanda Cifuentes Girard[1], Patrick Knight[2], and Gérard Hopfgartner[1]*

1. Life Sciences Mass Spectrometry, Department of Inorganic and Analytical Chemistry, University of Geneva, 24 Quai Ernest Ansermet, CH-1211 Geneva 4, Switzerland
2. Shimadzu Research Laboratory, Wharfside, Trafford Wharf Road, Manchester M17 1GP, United Kingdom.

* corresponding author author e-mail: gerard.hopfgartner@unige.ch

Figure S1: Chemical structures of drugs of abuse analytes.
Figure S2: Short LC-vDMS-MS configuration.
Figure S3: Overlaid compensation voltage plots for the isomeric pairs BZE/NCOC, ODT/NDT and THC/CBD at SV of 300 V.
Figure S4. Representative extracted ion chromatogram of urine sample acquired by LC-vDMS-SIM/MS.
Figure S5: Quantification of cocaine and metabolites.
Figure S6. Quantification of cannabinoids THC, CBD, and metabolites (THC-OH, THC-COOH, THC-COOH-GLU)
Table S1: Short LC-DMS-SIM/MS method performance for 8 drug of abuse and their metabolites in urine samples.
Table S2: LC-MRM/MS method performance for 8 drug of abuse and its metabolites in urine samples.
Table S3: LC-vDMS-SIM/MS method performance for 5 cannabinoids in urine samples.
Table S4. LC-MRM/MS method performance for 5 cannabinoids in urine samples.

A

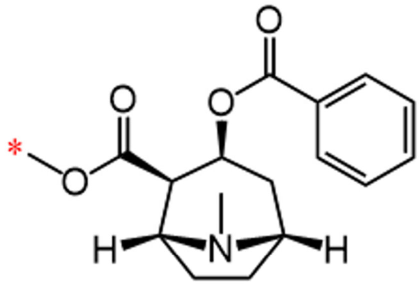

Cocaine (COC)
m/z 304.2
$C_{17}H_{21}NO_4$

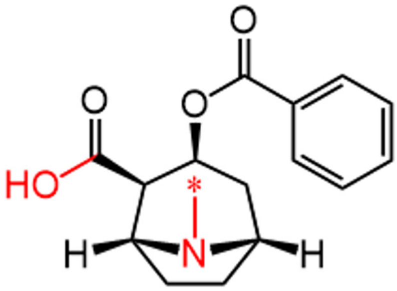

Benzoylecgonine (BZE)
m/z 290.2
$C_{16}H_{19}NO_4$

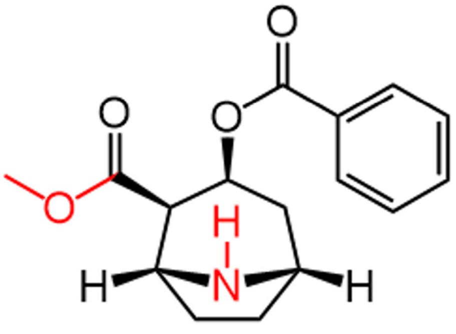

Norcocaine (NCOC)
m/z 290.2
$C_{16}H_{19}NO_4$

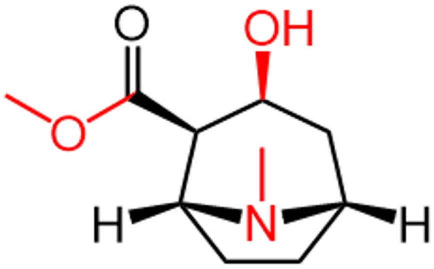

Ecgonine methyl ester (EME)
m/z 200.2
$C_{10}H_{17}NO_3$

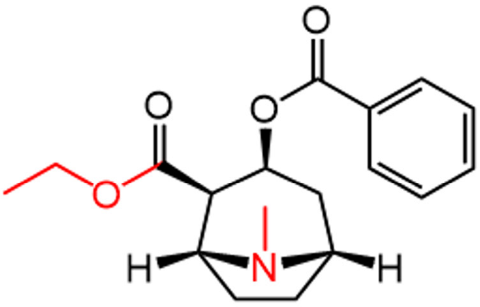

Cocaethylene (ECOC)
m/z 318.2
$C_{18}H_{23}NO_4$

B

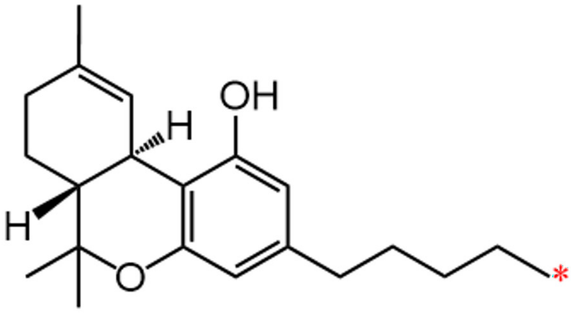

Δ^9-tetrahydrocannabinol (THC)
m/z 315.2
$C_{21}H_{30}O_2$

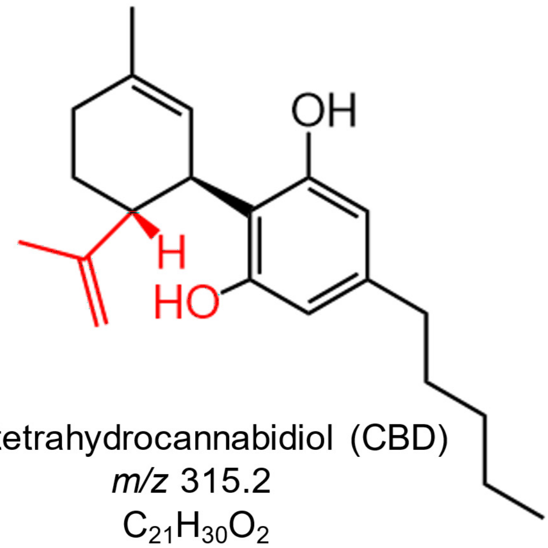

Δ^9-tetrahydrocannabidiol (CBD)
m/z 315.2
$C_{21}H_{30}O_2$

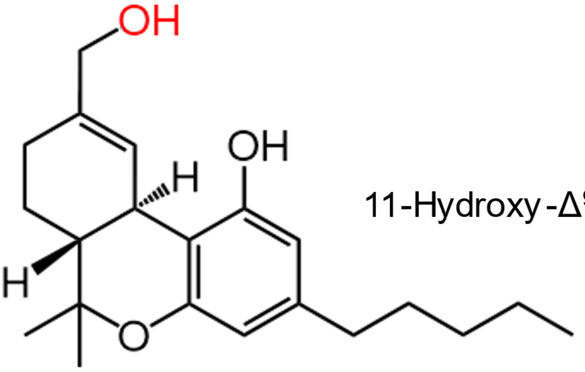

11-Hydroxy-Δ^9-tetrahydrocannabinol (THC -OH)
m/z 331.2
$C_{21}H_{30}O_3$

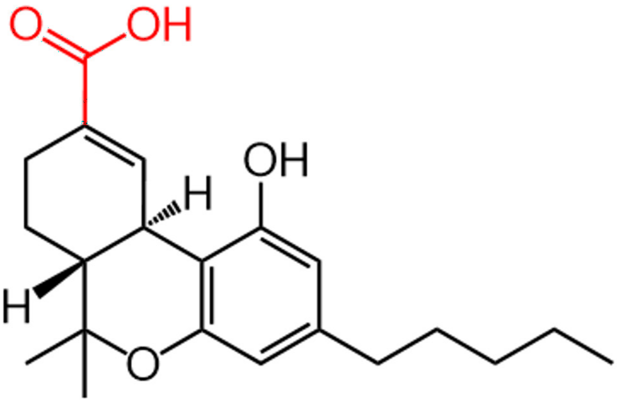

11-nor-9carboxy-Δ^9-tetrahydrocannabinol (THC -COOH)
m/z 345.2
$C_{21}H_{28}O_4$

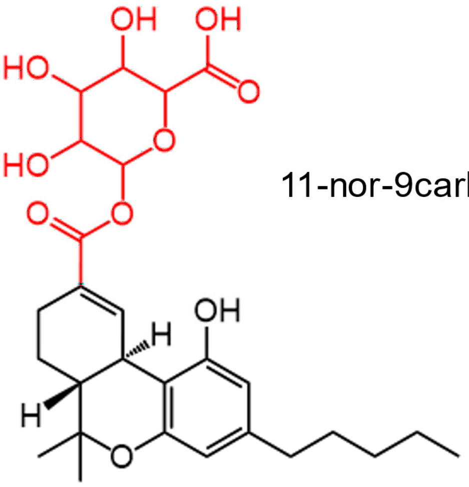

11-nor-9carboxy-Δ^9-tetrahydrocannabinol glucuronide (THC -COOH-GLU)
m/z 521.2
$C_{27}H_{36}O_{10}$

C

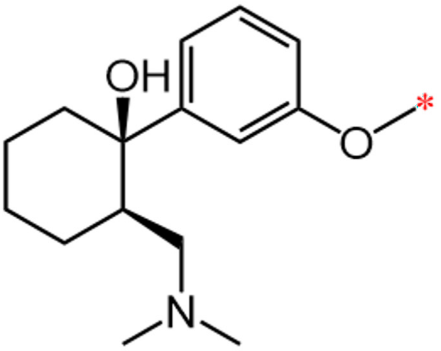

Tramadol (TRA)
m/z 264.2
$C_{16}H_{25}NO_2$

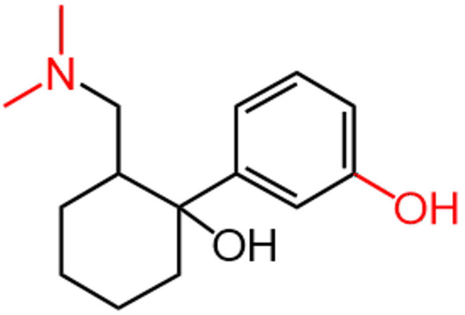

O-desmethyl-cis-tramadol (ODT)
m/z 250.2
$C_{15}H_{23}NO_2$

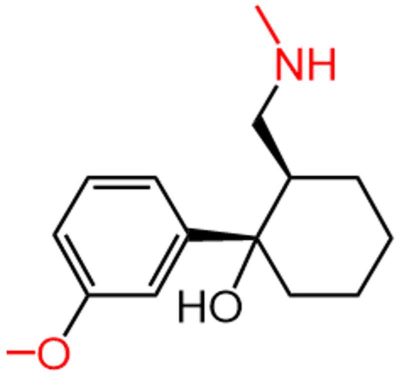

N-desmethyl-cis-tramadol (NDT)
m/z 250.2
$C_{15}H_{23}NO_2$

Figure S1. Chemical structures of drugs of abuse analytes. (A) cocaine and metabolites, (B) cannabinoids and metabolites and (C) tramadol and metabolites. Red stars indicate atoms isotopically labeled for internal standards (D_3).

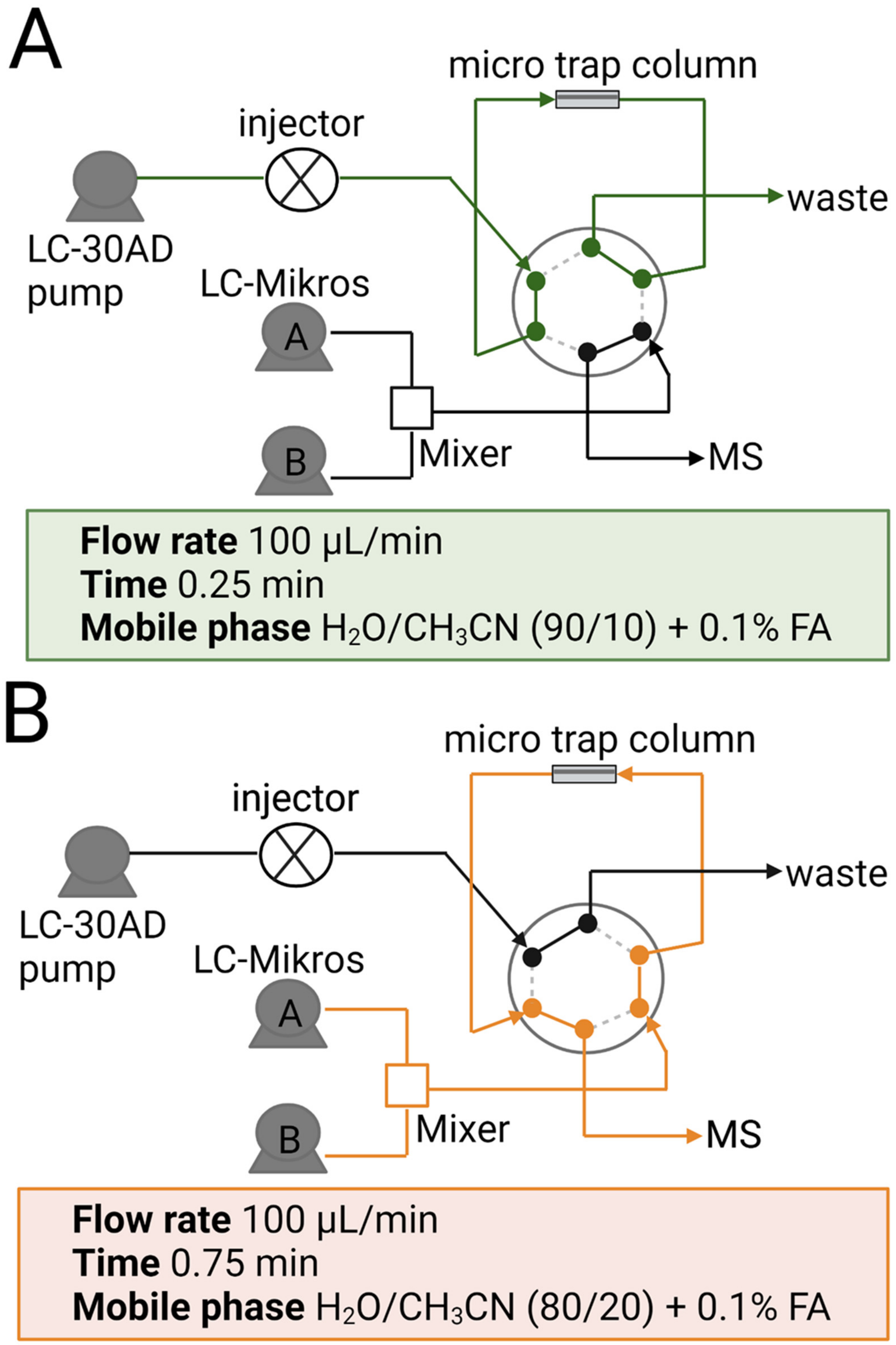

Figure S2. Short LC-vDMS-MS configuration. A short column Reprosil –Pur C18-AQ (5 µm, 10 mm x 0.5 mm i.d.) was used to analyse the samples in 2 steps: front-flush injection (A) and back-flush elution of analytes (B).

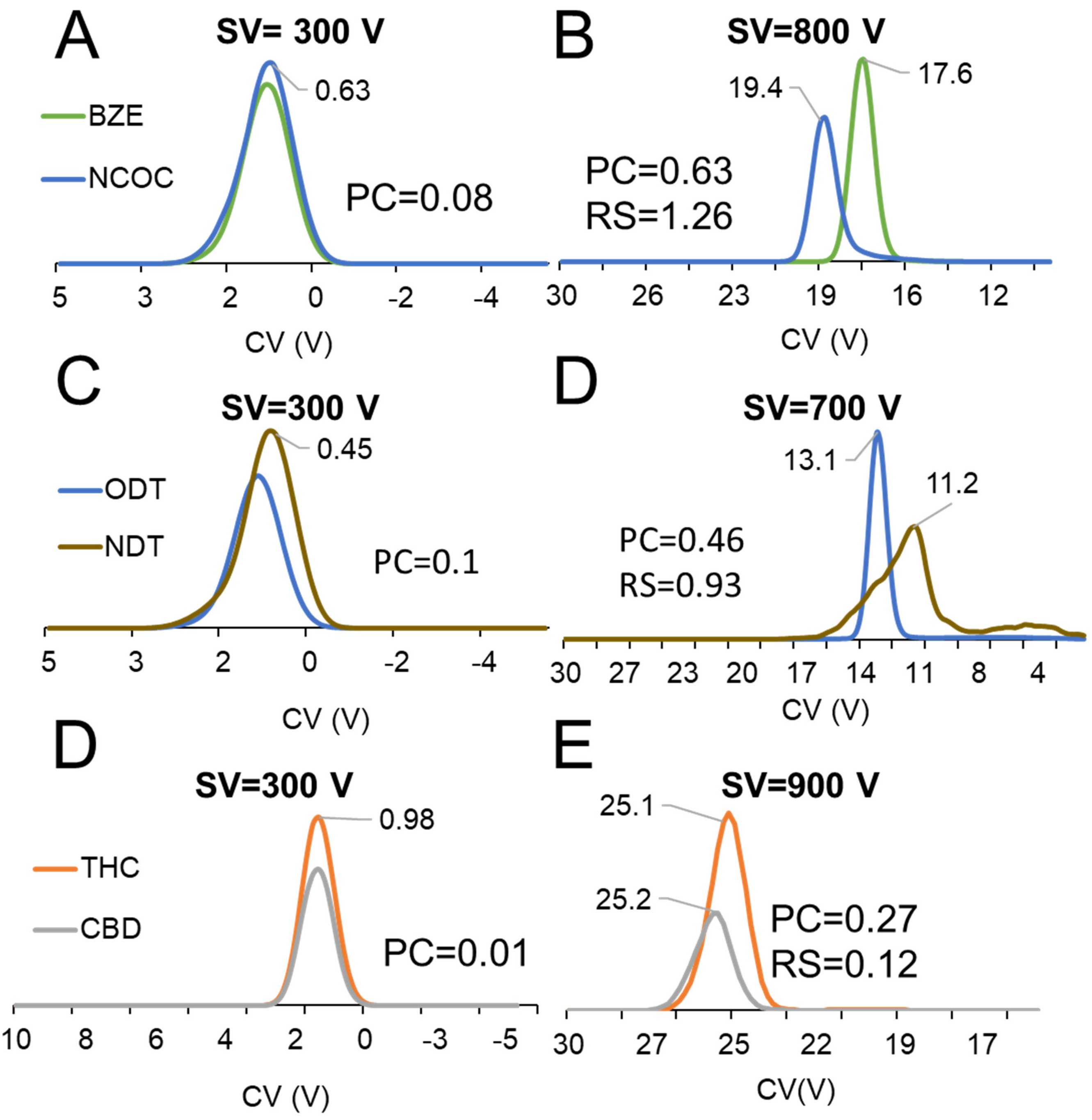

Figure S3. Overlaid compensation voltage plots for the isomeric pairs BZE/NCOC, ODT/NDT and THC/CBD at SV of 300 V (A, C, D) and 800 V (B, D, E). DMS cell temperature was 60°C, pressure 33 mbar, nitrogen as gas. The analytes were infused at 500 ng/mL at a flow rate of 8 µL/min (50/50 MeOH/H_2O, 0.1% formic acid).

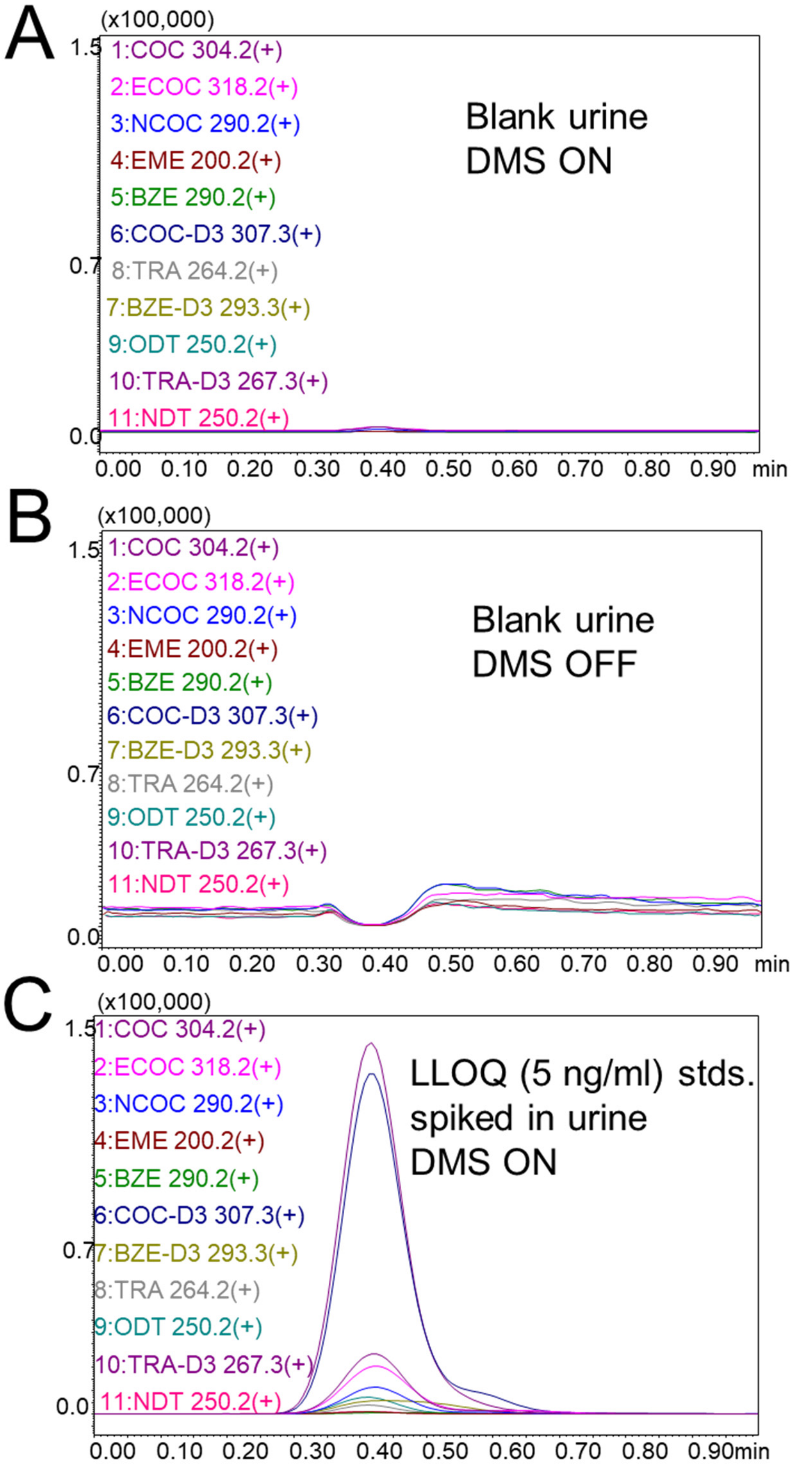

Figure S4. Representative extracted ion chromatogram of urine sample acquired by LC-vDMS-SIM/MS (A) with DMS ON (B) with DMS OFF and (C) spiked with standards at LLOQ (5 ng/ml) with DMS ON.

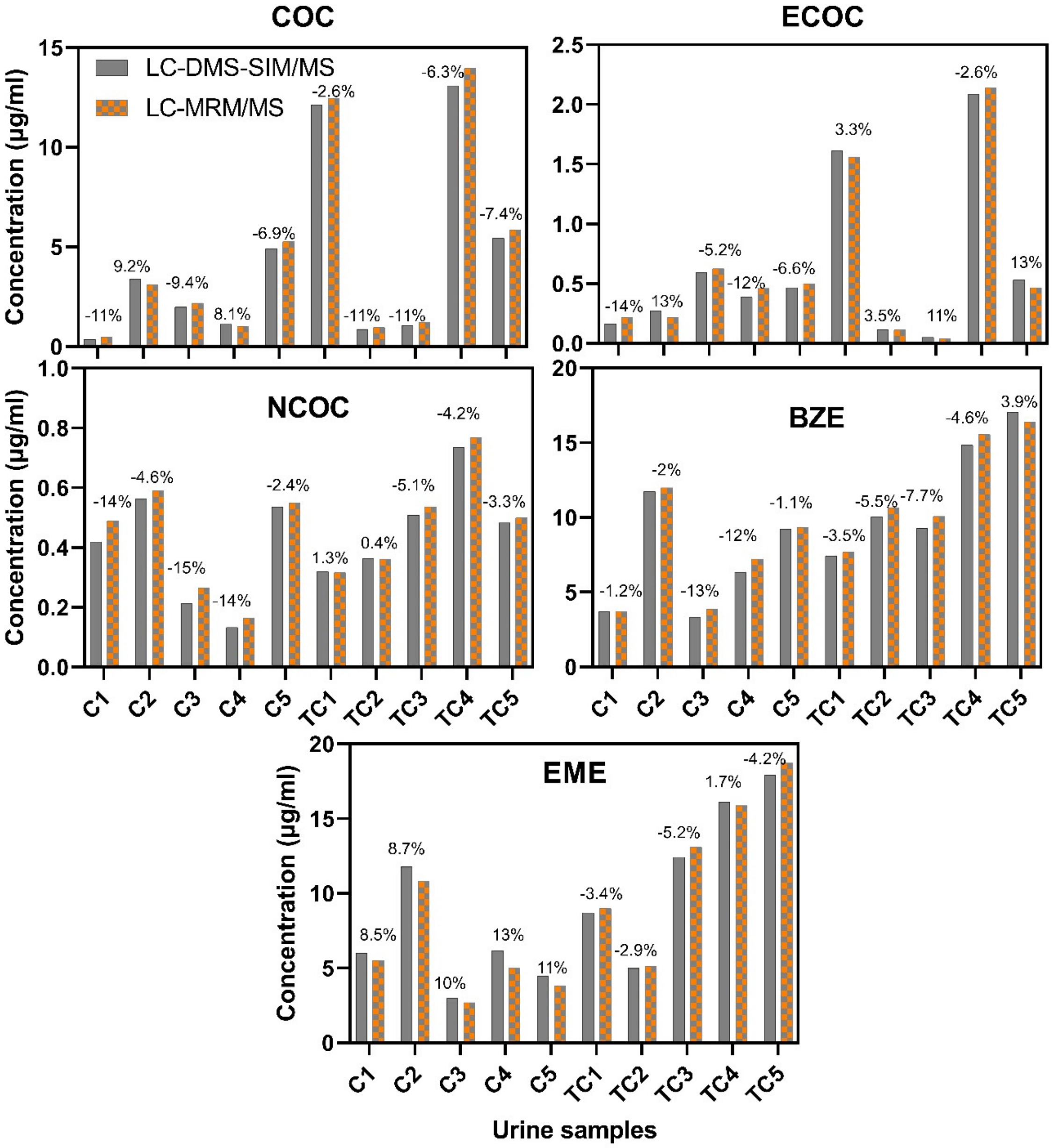

Figure S5. Quantification of cocaine and metabolites (ECOC, NCOC, BZE, EME) in ten urine samples collected from subjects who tested positive for THC and/or cocaine during roadside drug testing by short LC-vDMS-SIM/MS and LC-MRM/MS. Bias between the methods is presented as a %.

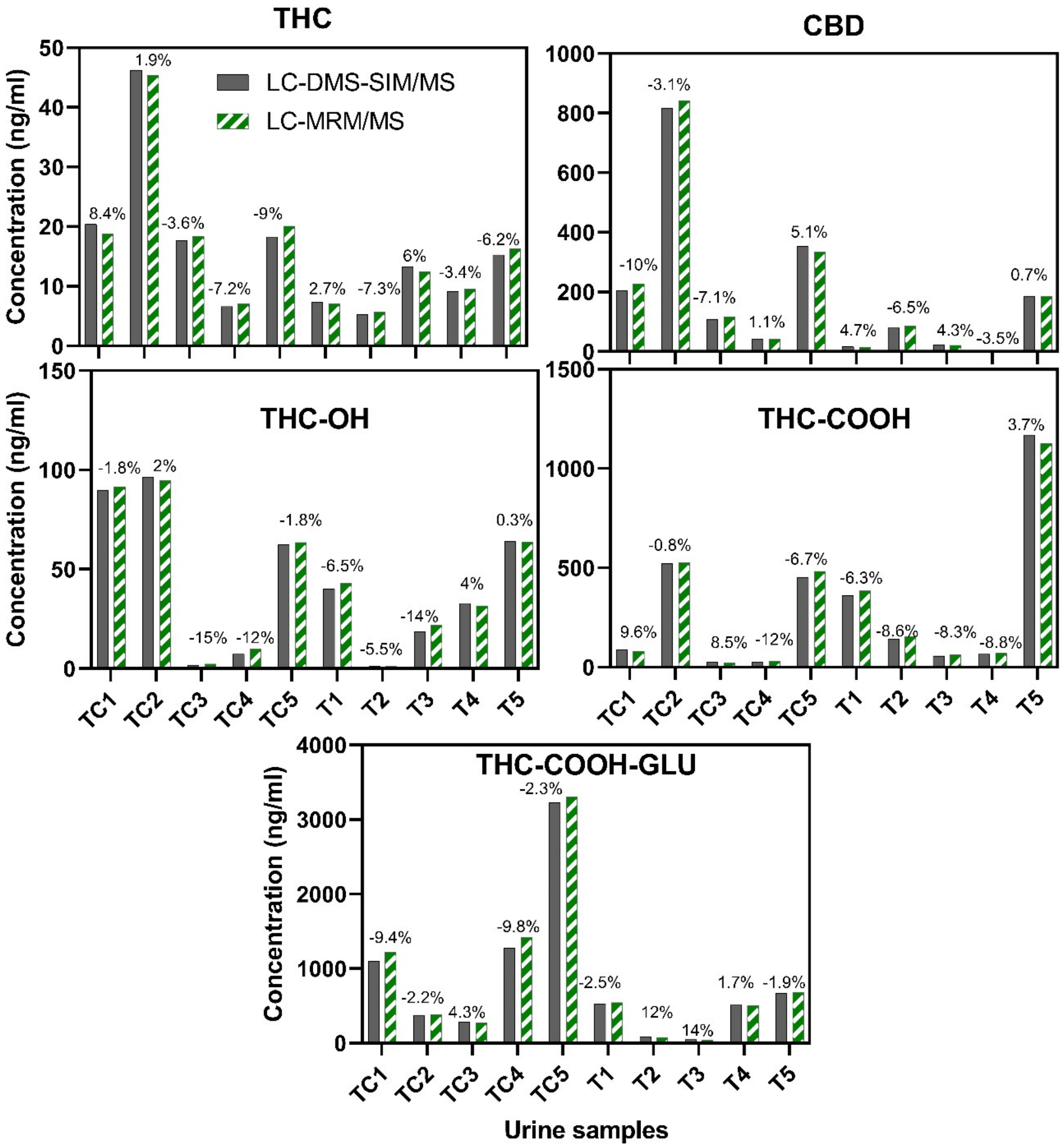

Figure S6. Quantification of cannabinoids THC, CBD, and metabolites (THC-OH, THC-COOH, THC-COOH-GLU) in ten urine samples collected from subjects who tested positive for THC and/or cocaine during roadside drug testing by short LC-vDMS-SIM/MS and LC-MRM/MS. Bias between the methods is presented as a %.

Table S1. Short LC-DMS-SIM/MS method performance for 8 drug of abuse and their metabolites in urine samples expressed in ng/ml. LLOQ: lower limit of quantification, LQC: Low Quality Control, MQC: Medium Quality Control, HQC: High Quality Control.

QC level		COC	ECOC	NCOC	EME	BZE	TRA	ODT	NDT
LLOQ	Conc.	5.000	5.000	5.000	5.000	5.000	5.000	5.000	5.000
	Average	5.650	5.350	5.100	5.000	5.350	5.200	5.250	5.300
	Accuracy%	113	107	102	100	107	104	105	106
	Precision%	7.4	9.2	9.7	1.2	10.6	3.1	5.3	9.2
	S/N	56	32	14	7	10	11	13	19
LQC	Conc.	15.00	15.00	15.00	15.00	15.00	15.00	15.00	15.00
	Average	16.05	15.00	15.75	16.65	15.75	15.15	14.57	16.20
	Accuracy%	107	100	105	111	105	101	97.1	108
	Precision%	5.8	4.9	5.8	12.2	8.1	6.8	6.3	4.8
	S/N	112	192	58	10	21	20	41	96
MQC	Conc.	3800	3800	3800	3800	3800	3800	3800	3800
	Average	3914	4104	3914	4066	4028	3952	3800	4028
	Accuracy%	103	108	103	107	106	104	100	106
	Precision%	4.6	4.1	8	1.9	5.7	4.9	8.2	4.3
	S/N	5332	1735	1195	73	133	904	382	1221
HQC	Conc.	7500	7500	7500	7500	7500	7500	7500	7500
	Average	7875	8400	7492.5	7357.5	7875	7440	7725	8250
	Accuracy%	105	112	99.9	98.1	105	99.2	103	110
	Precision%	1.3	0.9	3.5	7.8	6.5	6.7	3.5	2.2
	S/N	7498	1658	10120	163	495	1795	1850	2441

Table S2 LC-MRM/MS method performance for 8 drug of abuse and its metabolites in urine samples expressed in ng/ml. LLOQ: lower limit of quantification, LQC: Low Quality Control, MQC: Medium Quality Control, HQC: High Quality Control

QC level		COC	ECOC	NCOC	EME	BZE	TRA	ODT	NDT
LLOQ	Conc.	5.000	5.000	5.000	5.000	5.000	5.000	5.000	5.000
	Average	5.750	5.300	5.700	5.150	4.915	4.925	5.500	5.450
	Accuracy%	115	106	114	103	98.3	98.5	110	109
	Precision%	2.2	7.6	0.8	5.3	7.5	3.6	10.3	9.1
	S/N	3	3	4.3	4	8.9	4	3	8
LQC	Conc.	15.00	15.00	15.00	15.00	15.00	15.00	15.00	15.00
	Average	16.20	15.60	14.54	14.78	15.15	16.20	16.65	15.60
	Accuracy%	108	104	96.9	98.5	101	108	111	104
	Precision%	8	1.7	3.3	6.2	10	3.9	9.5	5.7
	S/N	40	35	689	15	747	23	397	10
MQC	Conc.	3800	3800	3800	3800	3800	3800	3800	3800
	Average	4142	4218	3914	3876	4180	4066	4142	4104
	Accuracy%	109	111	103	102	110	107	109	108
	Precision%	5.3	3.3	1	3.9	3.4	5.3	6.7	3.4
	S/N	3787	880	1670	85	1219	541	2023	4388
HQC	Conc.	7500	7500	7500	7500	7500	7500	7500	7500
	Average	7305	7800	7575	8100	8250	8175	8025	8025
	Accuracy%	97.4	104	101	108	110	109	107	107
	Precision%	3.2	1.8	1.5	5.8	2.9	2.9	3.2	2.2
	S/N	7575	1872	6083	190	3067	1024	5758	6867

Table S3. LC-vDMS-SIM/MS method performance for 5 cannabinoids in urine samples expressed in ng/ml. LLOQ: lower limit of quantification, LQC: Low Quality Control, MQC: Medium Quality Control, HQC: High Quality Control

QC level		THC	THC-OH	CBD	THC-COOH	THC-COOH-GLU
LLOQ	Conc.	1.000	1.000	1.000	1.000	1.000
	Average	1.080	1.110	1.120	1.054	1.105
	Accuracy%	108	111	112	105	110
	Precision%	5.1	7.5	6.5	10.2	9.5
	S/N	3.17	3.6	8.3	3.9	3.2
LQC	Conc.	3.000	3.000	3.000	3.000	3.000
	Average	3.300	3.300	3.270	3.097	3.185
	Accuracy%	110	110	109	103	106
	Precision%	3.6	6.7	4.8	8.4	7.4
	S/N	6.75	8.21	13.39	9.7	10.2
MQC	Conc.	375.0	375.0	375.0	375.0	375.0
	Average	427.5	427.5	386.3	390.0	382.5
	Accuracy%	114	114	103	104	102
	Precision%	2.1	5.4	4.2	5.4	4.5
	S/N	502	769.2	159.4	222	85.3
HQC	Conc.	750.0	750.0	750.0	750.0	750.0
	Average	795.0	787.5	757.5	704.3	733.5
	Accuracy%	106	105	101	93.9	97.8
	Precision%	3.2	4.7	3.1	4.6	5.2
	S/N	742	1199	188.8	378	128.4

Table S4. LC-MRM/MS method performance for 5 cannabinoids in urine samples expressed in ng/ml. LLOQ: lower limit of quantification, LQC: Low Quality Control, MQC: Medium Quality Control, HQC: High Quality Control

QC level		THC	THC-OH	CBD	THC-COOH	THC-COOH-GLU
LLOQ	Conc.	1.000	1.000	1.000	1.000	1.000
	Average	1.040	1.098	1.062	0.985	0.995
	Accuracy%	104	110	106	98.5	99.5
	Precision%	5.8	6.7	7.8	9.2	10.4
	S/N	3.7	9.1	4.7	3.1	3.4
LQC	Conc.	3.000	3.000	3.000	3.000	3.000
	Average	3.152	3.060	3.272	2.991	3.124
	Accuracy%	105	102	109	99.7	104
	Precision%	2.8	5.1	6.8	7.3	8.5
	S/N	10	15	12	11	9.5
MQC	Conc.	375.0	375.0	375.0	375.0	375.0
	Average	420.2	393.8	386.3	393.8	386.3
	Accuracy%	112	105	103	105	103
	Precision%	3.1	3.2	4.4	4.5	5.9
	S/N	620	164	689	215	88.9
HQC	Conc.	750.0	750.0	750.0	750.0	750.0
	Average	787.5	749.5	765.5	779.5	757.5
	Accuracy%	105	100	102	104	101
	Precision%	2.2	2.5	2.7	2.3	3.2
	S/N	940	258	1089	388	156.7
